# Evaluating large language models for annotating proteins

**DOI:** 10.1093/bib/bbae177

**Published:** 2024-05-05

**Authors:** Rosario Vitale, Leandro A Bugnon, Emilio Luis Fenoy, Diego H Milone, Georgina Stegmayer

**Affiliations:** Research Institute for Signals, Systems and Computational Intelligence sinc(i) (CONICET-UNL), Ciudad Universitaria, Santa Fe, Argentina; Research Institute for Signals, Systems and Computational Intelligence sinc(i) (CONICET-UNL), Ciudad Universitaria, Santa Fe, Argentina; Research Institute for Signals, Systems and Computational Intelligence sinc(i) (CONICET-UNL), Ciudad Universitaria, Santa Fe, Argentina; Research Institute for Signals, Systems and Computational Intelligence sinc(i) (CONICET-UNL), Ciudad Universitaria, Santa Fe, Argentina; Research Institute for Signals, Systems and Computational Intelligence sinc(i) (CONICET-UNL), Ciudad Universitaria, Santa Fe, Argentina

**Keywords:** large language models, protein annotations, transfer learning, protein families

## Abstract

In UniProtKB, up to date, there are more than 251 million proteins deposited. However, only 0.25% have been annotated with one of the more than 15000 possible Pfam family domains. The current annotation protocol integrates knowledge from manually curated family domains, obtained using sequence alignments and hidden Markov models. This approach has been successful for automatically growing the Pfam annotations, however at a low rate in comparison to protein discovery. Just a few years ago, deep learning models were proposed for automatic Pfam annotation. However, these models demand a considerable amount of training data, which can be a challenge with poorly populated families. To address this issue, we propose and evaluate here a novel protocol based on transfer learningṪhis requires the use of protein large language models (LLMs), trained with self-supervision on big unnanotated datasets in order to obtain sequence embeddings. Then, the embeddings can be used with supervised learning on a small and annotated dataset for a specialized task. In this protocol we have evaluated several cutting-edge protein LLMs together with machine learning architectures to improve the actual prediction of protein domain annotations. Results are significatively better than state-of-the-art for protein families classification, reducing the prediction error by an impressive 60% compared to standard methods. We explain how LLMs embeddings can be used for protein annotation in a concrete and easy way, and provide the pipeline in a github repo. Full source code and data are available at https://github.com/sinc-lab/llm4pfam

## INTRODUCTION

A current challenge in bioinformatics is the computational annotation of the immense protein universe. The annotation of the function of proteins is very demanding as a result of the high rate of current experimental data production [1]. For example, as of November 2023, there are more than 251 000 000 protein entries in UniProtKB (https://www.uniprot.org/); nevertheless only 570 157 (much less than 1%) have been manually curated by experts. This large gap between sequencing and annotation capabilities remains because it is easier and faster every time to generate experimental data, while, in contrast, the manual curation of results is very slow and time-consuming.

The most widely used tool for functional annotation of proteins is domain annotation. The Pfam(https://www.ebi.ac.uk/interpro/entry/pfam/) database is the repository of protein families and domains. Pfam has a list of well-known protein domain sequences called seed sequences from which a domain signature is drawn and is used for annotating new domains in novel proteins. Pfam uses BLAST [2] sequence similarity together with manually curated seed alignments of protein regions to obtain hidden Markov models (HMMs) profiles. The HMMs learn a sequence probabilistic representation of each family and are used to classify novel sequences [3]. In spite of the success of this current approach, there still remains a high percentage of unannotated proteins in Pfam that do not match any HMM profile. Currently, UniProt is growing at a much faster speed than its parallel Pfam coverage [4]

In many cases, this happens because it is difficult to estimate the HMM probabilities since in some families the examples available are just a few. HMM is trained as a generative model. This means that each HMM learns the probability distribution of one specific family, without using information of the others. Thus, the model for a given family is not able to use sequences from several families to learn inner patterns that might be shared among them. Generative models delve deep into the underlying data distribution. Each one aims to model the probability distribution of the training data for one class (family). This way, it can gain the ability to generate samples that closely resemble the training data. Discriminative models, in contrast, focus on learning the decision boundary that separates classes within data. Instead of modeling data distribution itself, they aim to model the discriminative information in the training data. Thus, discriminative models only work with labeled data and are very suited for classification tasks since they can effectively capture the differences between classes.

Deep learning (DL) models have been recently proposed for this task [5]. DL models are trained in a discriminative manner, looking at the complete sets of training patterns at the same time. Thus, those models are capable of inferring inner patterns or hidden rules shared across families, which can be very useful when new sequences have to be annotated [6]. Nonetheless, in order to infer meaningful patterns, large amounts of annotated data are required by DL models. This can be a constraint in the case of scarce families, thus the alternative we propose in this protocol is the transfer of knowledge from already trained and large models.

Following the path in natural language processing (NLP), several large language models (LLMs) for protein representation have recently appeared [7–9], which, given a raw protein sequence, can calculate a feature vector (embedding) that encodes its representation [10]. Next, a predictive model can solve a downstream task by learning from the embeddings that are associated with specific target labels. This is how LLMs that have acquired knowledge from one task can make the transfer learning (TL) of this knowledge into another task [11].

Protein embeddings, in particular, have recently appeared [12]. In a recent review [13] 12 protein sequence representation learning methods were experimentally benchmarked regarding sequence similarities representation in the embedding space, Gene Ontology terms functional inference and protein domain prediction. Results showed that Evolutionary Scale Modeling (ESM) [6] and ProtTrans [14] were the best methods in the evaluated tasks.

We will show in this protocol how the task of protein domain annotation into families can be largely improved by using TL from protein LLMs, trained in an auto-supervised way (without requiring external annotations) from large-scale protein data [15]. In [16] we have already shown that TL effectively improved classical machine learning models for this task. In this protocol we show and explain in detail how to combine TL with DL models for this task. We evaluate several recent LLMs in the same TL schema to assess their performance.

## TL AND LLMs FOR PROTEINS

### TL for proteins

TL is a machine learning technique where there is a transfer of the knowledge learned from a general task into a very specific task [17, 18]. It is a procedure where some knowledge that is acquired in one scenario can be taken advantage of in order to achieve better generalization in another one [19, 20]. The ability to learn knowledge from one task to solve related problems in other tasks has demonstrated that TL overcomes the limitation of standard DL models regarding the need of a very large amount of labeled data for training, specially when the source domain have large data availability and the corresponding task can be defined easily [21–23].

**Figure 1:**
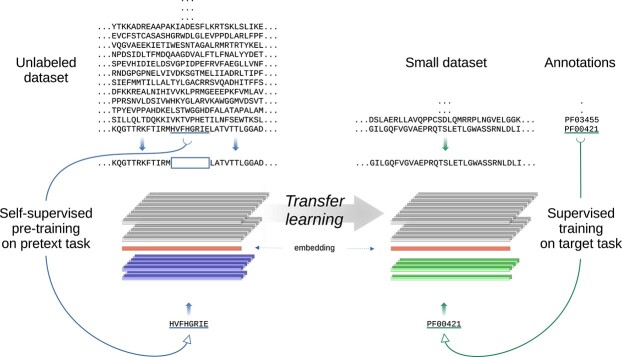
TL is an approach where a large model is trained on a generic task (left), and the knowledge obtained is afterwards transferred to be used for a specific task (right). The generic task in this protocol is protein sequence reconstruction by self-supervised learning on UniProtKB unlabeled data. After this pre-training, a task-agnostic deep LLM is generated (in gray), whose output representation (blue) is discarded. The middle layers of the LLM (orange) are frozen and transferred to another specific neural network architecture, whose last layers are adapted through supervised learning on a small labeled dataset to provide new representations (green) for a target task, which in this case of Pfam annotation.

The TL protocol proposed in this article is shown in Figure 1. At the left, there is a self-supervised LLM, which was trained without labels and for the task of predicting masked small segments of the input sequence. This is called pre-training on a pretext task, which in this case is the reconstruction of a protein sequence. This model has several hidden deep layers providing internal representations (in gray) and a fully connected architecture for the output representation (in blue), which will be discarded later. In the example of this Figure, the protein sequence inputs the model with part of its amino acids empty (HVFHGRIF) and the model has to learn how to predict it. After pre-training, the layers of the LLM architecture are used for feature extraction on new sequences (in gray), that is, these layers are ‘transferred’ to another model in order to solve a different task. The LLM weights are usually frozen to avoid the well-known catastrophic forgetting [24–26]. In this second stage, shown at the right of Figure 1, another supervised model is trained on a small labeled dataset for a specific task, which, in this case, is Pfam annotation. In the example, the second model at the right (in green), has to learn how to assign each protein segment, from the LLM embedding (in orange), to a Pfam family (PF00421 in the example).

From a practical point of view, the way of using TL in a pipeline for Pfam domain prediction is the following. First, obtain the embeddings from an LLM for all the training proteins. With those protein embeddings and their corresponding Pfam domain label, use supervised learning to train a model. After training your own model, or using one of the already trained models that we provide, in order to assign a family to a test protein the embedding of the testing protein must be calculated using the sequence as input of the LLM, as for the training proteins. Then, the test embedding is passed through the model to obtain the Pfam prediction. For example, if we obtain the embedding for the protein J7S630_KAZNA and pass it through a trained model, the output class with the highest score will be MutS_III (PF05192), which corresponds to its annotated Pfam domain at coordinates 214–539(https://www.ebi.ac.uk/interpro/protein/unreviewed/J7S630/). Annotations close to this domain, like PF00488, will not be confused by the model and the second and third output classes could be, for example, the Pfam domains DUF488 and ORMDL, but with extremely low scores.

### L‌LMs for proteins

LLMs that produce protein (numerical) representations (i.e. embeddings) are becoming more and more required by the community nowadays. Since very recently UniProtKB provides embeddings additionally to standard protein annotations (https://www.uniprot.org/help/embeddings), but only for two model organisms (*Homo sapiens* and *Escherichia coli*) and SARS-CoV-2. However, no protocol has been published yet with clear indications on how to use them in practice for protein function annotation. Multiple protein embedding methods appeared in the last 5 years [27]. A recent review [13] stated that there are just a few LLMs that can clearly outperform all others, even on diverse tasks. In that comparative study, ESM and ProtTrans were the best methods for all the bioinformatics tasks evaluated. For example, to quantify the ability of the embedding methods to be used for Pfam functional domain prediction, the cosine similarity between vector embeddings with a k-nearest neighbor classifier was evaluated. In that test, ESM and ProtTrans showed to perform noticeably better than other methods, regardless of the number $k$ chosen.

ESM and ProtTrans were pre-trained on millions of unaligned sequences from UniProtKB. Both are based on a powerful general-purpose model architecture for representation learning that has out-performed deep recurrent and convolutional neural networks, named Transformer [28]. The most widely used method for pre-training is named BERT (Bidirectional Encoder Representations from Transformers), which was originally designed for NLP [29], where context within a text is used to predict masked words. The self-supervised learning is performed under the hypothesis that word semantics can be derived from context, thus the subjacent language is learnt from these predictions. In ESM and ProtTrans, the input tokens are amino acids instead of syllables, making an analogy between text and proteins. They learn meaningful per-residue encodings by following the masked language modeling objective [29]. In the context of obtaining good protein representations, the masked language modeling is based on randomly masking some of the residues in the sequence (analogously to words in a sentence) and predicting which residues should replace those masks. Models learn a per-residue embedding that encodes its ‘meaning’ within the sequence by observing and taking into account its context. Hidden dependencies between amino acids can be learnt by this procedure. At the end, the per-residue representation can be averaged to a per-protein sequence embedding, to be used afterwards in the downstream task.

**ESM** [6](https://github.com/facebookresearch/esm): this family of models were pre-trained following the BERT procedure, using sequences of amino acids as inputs. The first optimized Transformer model on this task was ESM1b, which was trained on a high diversity subset of the UniProtKB database, taking the representatives of each cluster with similarity cutoff of 50% (UR50/S). In the case of ESM1v [30], it has the same architecture as ESM1b but it was trained on UniRef90 and it is an ensemble of five models. The most recent model available is ESM2 [31], a general-purpose model with up to 15B parameters, being the largest language model of proteins to date. During its training, sequences were sampled from across $\approx $ 43 million UniRef50 clusters and from $\approx $ 138 million UniRef90 sequences, so that during training the model saw $\approx $ 65 million unique sequences.

**ProtTrans** [14] (https://github.com/agemagician/ProtTrans): this model was developed using several Transformers. Two auto-regressive models (Transformer-XL and XLNet) and four auto-encoder models (BERT, Albert, Electra and T5) were trained with more than 2000 million proteins and up to 393 billion amino acids from UniRef50 and BFD databases [32]. BERT, mentioned before, is the standard for TL. Albert is a simpler version of BERT with fewer parameters. Electra is composed of a generator and a discriminator network for improving efficiency in the pre-training task. T5 is an encoder-decoder model, which projects a source language to an embedding space and then generates a translation to a target language. Although there are several ProtTrans instances available, in this study, we have used those indicated as the best ones (ProtBert-BFD and ProtT5-XL-U50) by the authors in its paper [14].

## DATA AND EXPERIMENTAL SETUP

### Data

Publicly available Pfam v.32.0 data were used for the experiments as in [5]. The benchmark annotation task includes seed sequences from 17,929 families. In order to assure remote homology (low similarity between training and testing sequences), authors in [5] used single-linkage clustering at 25% identity within each family to build a clustered split of the Pfam-seed data (as suggested in [33]). The objective was to have an objective measure of how a model would perform in a realistic scenario with testing sequences that are not similar to the training ones. The clustered benchmark was chosen because if a large number of sequences are evolutionarily related, a random split would include training and testing sequences that are very close, and thus the model can memorize the training samples to classify the test one. Instead, in the clustered split all held-out test sequences are guaranteed to be far from the train set, avoiding overoptimistic results.

There are 1 339 083 sequences for training and 21 293 sequences for testing in the benchmark dataset. A development subset of 10% training data was used for early stopping. For all the data (training + testing) we have obtained the corresponding embeddings using the following pre-trained models:

- ESM-1b: esm1b_t33_650M_UR50S.
- ESM-1v: esm1v_t33_650M_UR90S_[1-5].
- ESM2: esm2_t33_650M_UR50D.
- ProtBert-BFD: Rostlab/prot_bert_bfd.
- ProtT5-XL-U50: Rostlab/prot_t5_xl_half_uniref50-enc.

### Experimental setup

In this protocol, we compared the following methods for the Pfam families classification task. Models without TL (HMM, BLASTp, ProtCNN and ProtENNas reported in [5]); versus the following ML models with TL (that is, with input embeddings):

**KNN**: a standard $k$-nearest neighbor ($k$NN) classifier [34] with $k=1$ for the neighborhood and Euclidean distance between embeddings. Embeddings are averaged across residues.

**MLP**: a multi-layer perceptron (MLP) neural network [35] was implemented with a classical architecture of 4 hidden layers of size 500, 100, 100 and 1000, respectively. The hyperparameters have been determined in previous experiments following [5], with a small development subset of the clustered dataset that includes 21,510 sequences well-separated from the train and test sets. We have performed a grid search on the following hyperparameters: the number of hidden layers (2, 3, 4), and the number of neurons in each layer (100, 500, 1000, 5000, 10000). Optimization was performed with Adam solver and backpropagation with cross entropy loss, using early-stopping with patience = 5 epochs in the development subset. Embeddings are averaged across residues to obtain the features input.

**MLP-E**: an ensemble of five MLP models as described above regarding architecture, training options and optimization solver. The ensemble used a majority vote algorithm to determine the output class, where the most voted element among each classifier prediction determines the output class.

**CNN**: a convolutional neural network (CNN) adapted from [5]. in the input, a 1D convolutional layer has input size according to each per-residue embedding (1280 in the case of ESM, 1024 in the case of ProtTrans), with 1100 filters and kernel size 9. Then five residual network blocks (ResNets) with 0.5 factor in the bottleneck units and dilated convolutions are included. The output is a fully connected layer that provides the per class scores. Cross-entropy loss and Adam optimizer are used during training.

**CNN-E**: an ensemble of five CNNs using the maximum activation of the average scores, where each model was trained from a different random initialization of parameters.

## RESULTS

### Comparison between no TL and TL approaches for Pfam families classification

Table 1 shows, for each classification method without TL (in rows), the error rate calculated as the percentage and the absolute number of errors as the difference between predicted family and golden standard family for all test sequences. This table reproduces the results reported in [5], for the same dataset and train/test partitions. In this work we used error rate and number of errors in order to be able to compare the results directly with [5]. However, the error rate is just one possible way of measuring performance, which has the limitation of not taking into account false negatives. HMM and BLASTp use the raw sequences, and ProtCNN and ProtENN are fed with one-hot encoded inputs. The best value (lowest classification error) is indicated in bold. It can be seen that in this table the ensemble of CNNs using one-hot encoding, that is without TL, achieves the best error rate of 12.20% corresponding to 2590 errors.

**Table 1:** Pfam families classification without TL

No-TL	Error rate	#errors
HMM	18.10%	3844
BLASTp	35.90%	7639
ProtCNN	27.60%	5882
ProtENN	**12.20%**	**2590**

Table 2 shows the results of the classifiers using TL, that is, using inputs encoded with different embeddings, from top to bottom: ESM1b, ESM1v, ESM2, ProtBert-BFD and ProtT5-XL-U50. The results are reported as error rate and number of errors. several runs were made for each model, with very small variations (below 0.01). The best result for each embedding is indicated in bold. The reproducibility of the results in Table 2 can be verified at the github repo https://github.com/sinc-lab/llm4pfam.

**Table 2:** Pfam families classification with TL

***ESM1b***	Error rate	#errors
KNN	15.16%	3229
MLP	35.23%	7501
CNN	16.66%	3548
MLP-E	21.93%	4669
CNN-E	**9.56%**	**2035**
***ESM1v***		
KNN	21.33%	4541
MLP	41.41%	8818
CNN	18.79%	4000
MLP-E	27.33%	5819
CNN-E	**10.94%**	**2329**
***ESM2***		
KNN	15.55%	3311
MLP	30.88%	6576
CNN	17.48%	3721
MLP-E	18.10%	3854
CNN-E	**7.67%**	**1634**
**ProtBert-BFD**		
KNN	39.49%	8408
MLP	48.16%	10254
CNN	26.97%	5743
MLP-E	34.64%	7375
CNN-E	**15.08%**	**3211**
**ProtT5-XL-U50**		
KNN	8.63%	1838
MLP	26.32%	5604
CNN	16.61%	3537
MLP-E	15.08%	3211
CNN-E	**7.28%**	**1550**

If all embeddings are compared, ProtT5-XL-U50 and ESM2 are those that allow obtaining the best results, that is, the lowest errors. Both embeddings, when used in combination with an ensemble of DL models, achieve the lowest error rate (7.28 and 7.67%, respectively). It is very interesting to note here that the use of TL (ProtT5-XL-U50) combined with a simple KNN can achieve better results (8.63%) than the best result of Table 1 (12.20%), which is an ensemble of DL models.

If models are analyzed, it can be stated that in all cases, the use of TL makes them achieve competitive results in comparison to Table 1. As stated before, a simple KNN with ProtT5-XL-U50 achieves a very low error rate. In the case of MLP, it has the lowest error when combined with ProtT5-XL-U50. The single CNN model achieves very similar and good results with both ESM1b and ProtT5-XL-U50. Regarding the ensembles of MLP, as it could be expected, achieve a better result than a single MLP in all cases. Regarding CNN and CNN-E, they are the best models in each table, being CNN-E with ProtT5-XL-U50 and CNN-E with ESM2 the ones with the lowest error rate in all the comparisons made.

In summary, if the best model not using TL (ProtENN) and the best models using TL (CNN-E with ProtT5-XL-U50 and CNN-E with ESM2) are compared, the last ones reach the best global results. It can be noticed that the error rates of CNN-E with TL are almost half the error of the ensemble of DL models without TL in Table 1. This confirms the hypothesis of this study, that protein family classification could be greatly enhanced with self-supervised learning by transferring LLM representations of protein sequences already learned without requiring annotations from large-scale protein data. Moreover, if the error rate of standard Pfam classification method (HMM, 3844 errors) is compared against the best performing method with TL (1550 errors), it can be stated that the TL approach could improve the actual classification models by 60%.

### Detailed analysis for families with few samples

**Figure 2:**
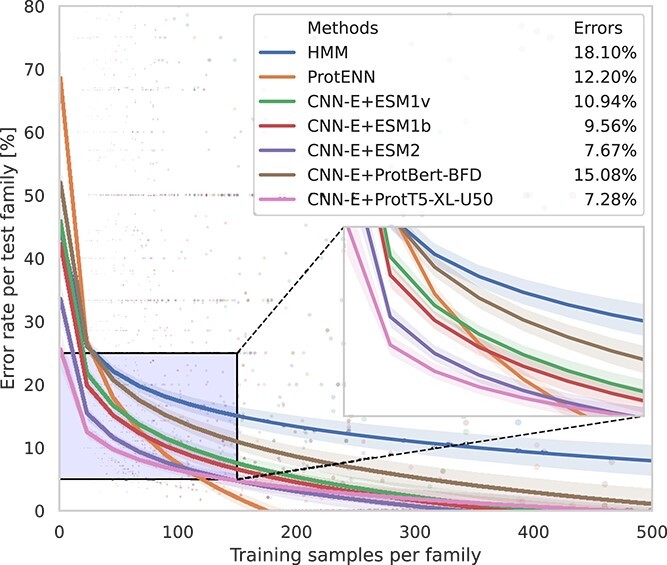
Linear regression (lines) of the error rates (dots) per test families classification for HMM, ProtENN (one-hot input) and each embedding here evaluated with the CNN-E classifier. The *x*-axis shows the number of samples at each family in the training partition, from left (fewl) to right (many samples), showing only up to 500 training samples per family. The legend indicates the error rate for all test families. The regression models were fitted with the number of samples per family in logarithmic scale.

Figure 2 shows the error rate for HMM, ProtENN and each embedding with the best model (CNN-E) for Pfam families classification. HMM and ProtENN were trained and tested by us to reproduce the results of Table 1. The x-axis indicates the number of samples of each family in the training partition, from left (few) to right (many samples). It can be clearly seen here that where there are just a few training samples of a family, all methods have a very large error rate. At the extreme left, 1 training sample per family, ProtT5-XL-U50 and ESM2 with the CNN ensemble have the lowest error. As training samples of the families increase, all methods decrease the error rate. HMM is, in any case, the method with more errors. The number of classification errors is large until a minimum number of sequences are present in the training set. In the case of ProtENN, it has the largest error rates when there are very low training patterns for each family, as expected for DL models without TL. However it should be noted that ProtENN outperforms other methods given a sufficient number of training samples (at least 200 training samples per family). All methods decrease the prediction error below 20% when there are at least 100 samples per family in training. Notably, the best performing embedding methods (ProtT5-XL-U50 and ESM2) can achieve that error rate in the case of very low populated families, with less than 30 samples per family (see the zoom in Figure 2).

A deeper analysis was made for those families with less than 30 examples in the training set (1398 families). The error rate was calculated for the corresponding (4420) testing sequences of those families. HMM and ProtENN without TL were trained and tested by us to reproduce the results of Table 1. The HMM models were evaluated with the same testing sequences, achieving a 49.69% error rate. The same procedure was done with ProtENN, achieving a 36.60% error rate. In the case of the CNN-E with TL, the error rates achieved were: ProtBertBFD 32.84%, ESM1v 27.59%, ESM1b 25.58%, ESM2 20.34% and ProtT5-XL-U50 16.33%. This detailed analysis also confirms the hypothesis of this study: that the TL approach can effectively improve the Pfam prediction rates, even in the case of families with very low training examples.

## CONCLUSIONS

In this protocol, we implemented TL to boost protein families classification in the Pfam database. Results achieved on a clustered testing partition showed that even the most simple machine learning classifier can achieve better results than models without TL. The results indicate that using TL for protein families classification can impressively reduce the prediction error in comparison to standard methods and to DL models without TL. The outcomes suggest that TL is a feasible and effective solution for boosting protein classification, and it will become part of future protein annotation tools.

This protocol has shown how, instead of building a particular embedder, it is more advantageous to recycle all computation time already spent by the available protein LLMs. We conclude that using TL in combination with supervised classifiers trained on small annotated sets can have a significant impact on performance.

**Key Points**

- We have shown here a protocol based on transfer learning for protein annotation, which involves self-supervised learning on a large unlabeled dataset.
- Numerical embedding for each sequence can be easily obtained from LLMs and used afterwards with supervised learning on a small labeled dataset.
- Transfer learning for protein families classification can impressively reduce the prediction error by more than half compared to standard methods.
- Transfer learning is a viable and effective solution for improving protein classification.

## FUNDING

This work was supported by ANPCyT (PICT 2018-3384, PICT 2022-0086), Agencia Santafesina de Ciencia, Tecnología e Innovación (PEICA-2022-059, PEIC I+D 2022 075) and UNL (CAI+D 2020 115).

## Author Biographies

**Rosario Vitale** is a student of Informatics Engineering at Universidad Nacional del Litoral (UNL). She currently holds a research fellowship under the supervision of G. Stegmayer at the Research institute for signals, systems and computational intelligence, sinc(i), Santa Fe, Argentina.

**Leandro A. Bugnon** is research assistant at sinc(i) since 2020. His research interests include automatic learning, pattern recognition, signal and image processing, with applications to bioinformatics, biomedical signals and affective computing.

**Emilio L. Fenoy** is a bioinformatician with experience in immunoinformatics and proteomics. He currently holds a postdoctoral position at the Research Institute for Signals, Systems and Computational Intelligence, sinc(i), Santa Fe, Argentina.

**Diego H. Milone** is a full professor in the Department of Informatics at Universidad Nacional del Litoral (UNL) and principal research scientist at CONICET. His research interests include statistical learning, signal processing, neural and evolutionary computing, with applications to biomedical signals and bioinformatics.

**Georgina Stegmayer** is a professor in the Department of Informatics at Universidad Nacional del Litoral (UNL), and Principal Researcher at the sinc(i) Institute, National Scientific and Technical Research Council (CONICET), Argentina. She is the Leader of the Bioinformatics lab at sinc(i). Her current research interest involves machine learning, data mining and pattern recognition in bioinformatics.

**sinc(i)** is a research institute for Signals, Systems and Computational Intelligence. Research at sinc(i) aims to develop new algorithms for machine learning, data mining, signal processing and complex systems, providing innovative technologies for advancing healthcare, bioinformatics and human-computer interfaces. The sinc(i) was created and is supported by the two major institutions of highest education and research in Argentina: the National University of Litoral (UNL) and the National Scientific and Technical Research Council (CONICET).
